# Enhancement of tribological behavior and microhardness of AISI H13 tool steel by electrochemical boriding

**DOI:** 10.1038/s41598-025-28422-7

**Published:** 2025-12-08

**Authors:** A. Mourad, A. A. Mahdy, E. S. Mosa, A. Kandil, M. A. Elhelaly

**Affiliations:** 1 Mining, Metallurgy, and Petroleum Engineering Department, Faculty of Engineering, Al-Azhar University, Nasr City, Cairo, 11371 Egypt; 2 Heat Treatment Department, Tabbin Institute for Metallurgical Studies, Cairo, Egypt; 3 Intercairo Company for Aluminum Industry, 6th October, Giza, Egypt

**Keywords:** Hot work tool steel, Electrochemical boriding, Iron boride coating, Microhardness, Activation energy, Tribological behavior, Materials chemistry, Metals and alloys

## Abstract

Iron boride coatings were developed on AISI H13 hot work tool steel using the electrochemical boriding method at temperatures of 850, 950, and 1050 °C for durations of 2, 4, and 6 h. The process was conducted at a current density of 200 mA/cm^2^, utilizing a powder mixture containing 22.5 wt.% ferroboron (Fe-B), 70 wt.% borax (Na_2_B_4_O_7_), and 7.5 wt.% ammonium chloride (NH_4_Cl). The obtained coatings were examined using light microscopy (LM), scanning electron microscopy with energy-dispersive spectroscopy (SEM/EDS), and X-ray diffraction (XRD). Metallographic analysis revealed a distinct saw-tooth shaped interface between the boride layer and the underlying transition zone, which was consistent and uniform across the examined area. XRD results revealed the formation of a dual-phase boride layer (FeB/Fe_2_B) with traces of chromium and vanadium borides. The kinetics of the boriding process were evaluated using the classical parabolic growth law, demonstrating a parabolic relationship between boride layer thickness and treatment time. The activation energy required for boron diffusion throughout the boride layer was determined to be 168.4 kJ/mol. Additionally, the microhardness and wear rate were evaluated. The boride layer reached a thickness up to 252 µm and exhibited a microhardness of 1956 ± 67 HV_0.05_, representing an increase of over 300% compared to the quenched and tempered specimens, which had a microhardness of 543 ± 8 HV_0.05_. The findings demonstrated that the phase composition and thickness of the boride coatings are strongly influenced by the immersion time and processing temperature.

## Introduction

Boronizing is a commonly employed thermochemical surface treatment designed to enhance the wear and friction properties of metallic materials. This process produces an exceptionally hard surface, with hardness values ranging from 1450 to 2000 HV, significantly improving the wear resistance of metal surfaces. As a result, boronizing offers notable advantages over traditional thermochemical methods such as carburizing and nitriding^1–3^.

Various boriding techniques have been developed, including pack cementation^4–6^, salt bath^7^, slurry coatings^8^, plasma-assisted methods^9,10^, and more recently, electrochemical boriding (EB)^11–13^. In particular, EB in molten salt baths has emerged as a promising alternative to conventional processes. This method facilitates the formation of atomic boron on the substrate through current-driven electrochemical reactions^14,15^, offering advantages such as lower processing temperatures, shorter treatment times, and improved energy efficiency^16,17^.

AISI H13 tool steel is widely used in die casting, hot extrusion, and plastic injection molding due to its excellent thermal fatigue resistance, high hardness, and good toughness. To further enhance its performance under severe service conditions, several studies have applied boriding to this steel, leading to the formation of hard, wear-resistant boride layers on its surface^18–20^. For example, Genel^18^ examined the boriding kinetics of H13 steel in a solid medium and modeled the layer thickness growth using the parabolic law. Krelling et al.^19^ investigated the tribological performance of H13 steel borided with various agents and highlighted differences in friction and wear behavior. López-Perrusquia et al.^20^ characterized the morphology and brittleness of boride layers, emphasizing the influence of processing conditions.

Subsequent research expanded upon the understanding of diffusion mechanisms and tribological improvements. Nair et al.^21^ applied simultaneous austenitizing and multi-directional boriding, achieving improved surface properties. Morón et al.^22^ evaluated friction and wear behaviors under dry and lubricated conditions. Karakaş et al.^23^ analyzed boride layer growth using nano-sized powders, while Ortiz-Domínguez et al.^24^ focused on diffusion modeling and microstructural characterization. These studies underline the importance of optimizing boriding parameters such as temperature, time, and boriding medium.

Electrochemical boriding has gained attention for its ability to produce thicker coatings in shorter durations than conventional methods^25–28^. Matiasovsky et al.^26^ observed the formation of FeB and Fe_2_B phases above critical current density and suggested electrolyte temperature as the key factor controlling layer growth. In contrast, Seger et al.^27^ proposed that electrochemical parameters rather than thermal effects primarily govern the formation of single-phase Fe_2_B layers.

Despite the growing body of literature on the boriding of AISI H13 steel, comparative studies on the electrochemical boriding process using molten salt electrolysis remain limited. Furthermore, a comprehensive understanding of how parameters such as treatment duration and electrolyte temperature influence layer structure, thickness, microhardness, and wear behavior is still developing.

Therefore, the objective of this study is to investigate the electrochemical boriding of AISI H13 tool steel using molten salt electrolysis. The influence of processing parameters on layer growth, tribological performance, and surface hardness is systematically analyzed, contributing new insights into the efficiency and applicability of this surface engineering approach.

## Experimental work

### Materials and specimens preparation

In this study, Hot work tool steel (AISI H13) specimens were cut to a cylindrical shape with dimensions of Ø 12 mm (diameter), 20 mm (length) with a 2 mm hole in center for hanging purposes, used as a substrate. The chemical compositions of AISI H13 are provided in Table 1.

**Table 1:** The chemical compositions of AISI H13 hot work tool steel in wt. %.

Element	C	Si	Mn	Cr	Mo	V	Fe
Wt.%	0.33	1.03	0.43	4.58	1.27	0.83	Balance

**Fig. 1:**
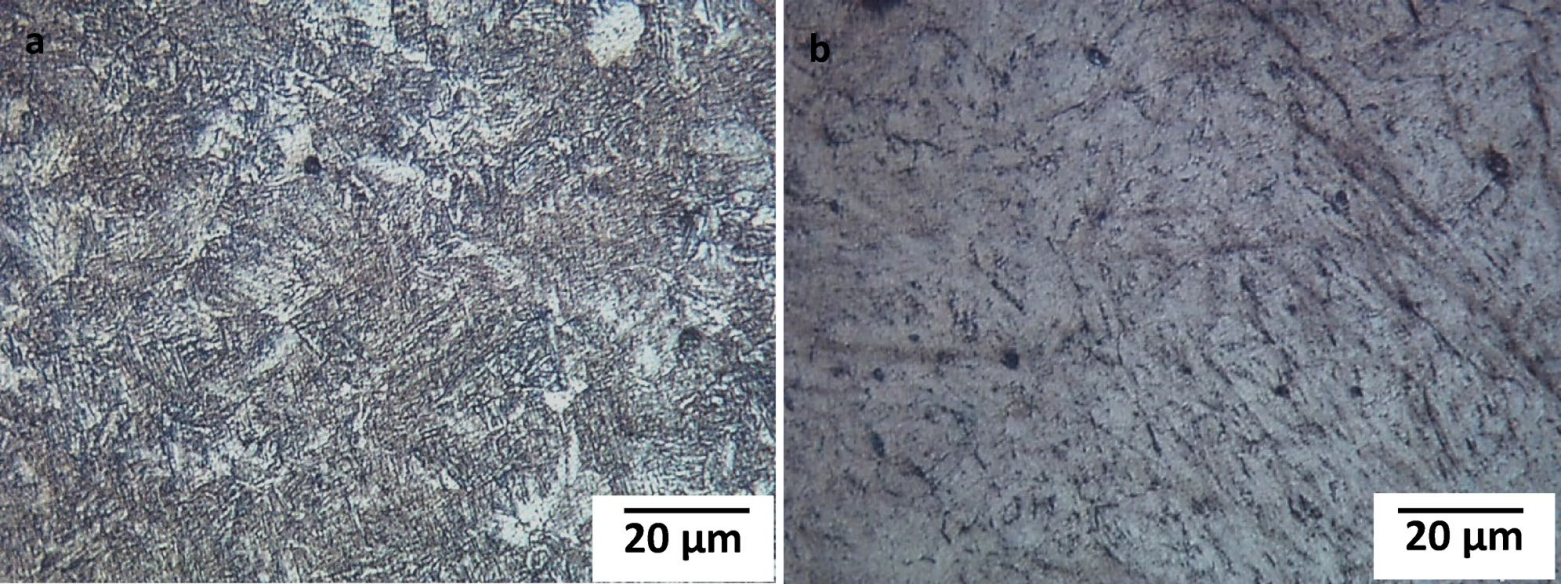
The microstructure of AISI H13 tool steel annealed (**a**), hardened (**b**).

As stated in the technical data sheet, the AISI H13 specimens were annealed at 850 °C for 2 h, resulting in a hardness of 28 HRC then hardened at 1030 °C for 0.5 h, followed by air quenching and tempering at 580 °C for 2 h. Prior to thermo-reactive deposition (TRD) treatment, the specimens were ground using silicon carbide papers from 180 to 1000 grits, ultrasonically cleaned in ethanol for 5 min and dried. The microstructures of annealed and hardened AISI H13 after being etched by nital 2% solution. Figure 1 shows the microstructure of the AISI H13 specimens before the TRD treatment. As shown in the Figure (1a), the microstructure of the annealed consists of fine spheroidal particles of carbides in a matrix of ferrite and bainite matrix in hardened.

### Growth of the coatings

The molten salt baths, shown in Table 2 used for boriding consisted of anhydrous borax (Na_2_B_4_O_7_), ferroboron (Fe-B) of particle size ≤ 100 µm containing 80 wt.% B, and ammonium chloride (NH_4_Cl), as the filler material, boron source, and chemical activator, respectively.

**Table 2:** Compositions of the salt bath mixtures (in wt. %).

Components	Fe-B	NH_4_Cl	Na_2_B_4_O_7_
Mass fraction	22.5%	7.5%	70%

The specimens prepared for the boriding process were positioned in cylindrical-shaped crucibles with dimensions (Ø80 mm, H 110 mm, T 10 mm) made of AISI 310 heat-resistant stainless steel. The crucibles were then placed inside an electric muffle furnace (Nabertherm, model KSL-1400X). Initially, borax was melted in a crucible, after which the other components were added to the salt bath. To ensure homogenization, the bath was heated to 800 °C for at least 1 h prior to the immersion of the specimens. The specimens were preheated at 350 °C for 10 min before being immersed in the prepared bath to minimize thermal shock and prevent surface cracking due to the sudden temperature gradient between the ambient sample and the high-temperature molten bath. This step also promotes the removal of any adsorbed moisture and volatile contaminants from the surface, ensuring better electrical contact and more uniform boron diffusion during electrochemical treatment. Additionally, preheating facilitates a smoother transition to the target process temperature, reducing residual stress in the treated layer^29^. The boronizing process was carried out at temperatures of 850, 950, and 1050 °C for durations of 2, 4, and 6 h, respectively.

The setting of current and voltage values used a power supply with a potentiometer to deliver a specific current (e.g., in Amperes) to the electrochemical cell. By regulating the input 220 V and the output 22–28 V you can adjust the output current. The current density is then calculated by dividing the total current by the surface area of the sample (cathode). according to Faraday’s first law, the amount of boron diffused is directly proportional to the quantity of electricity passed. the voltage across the electrodes is fixed, and the current will vary depending on the output volt.

**Fig. 2:**
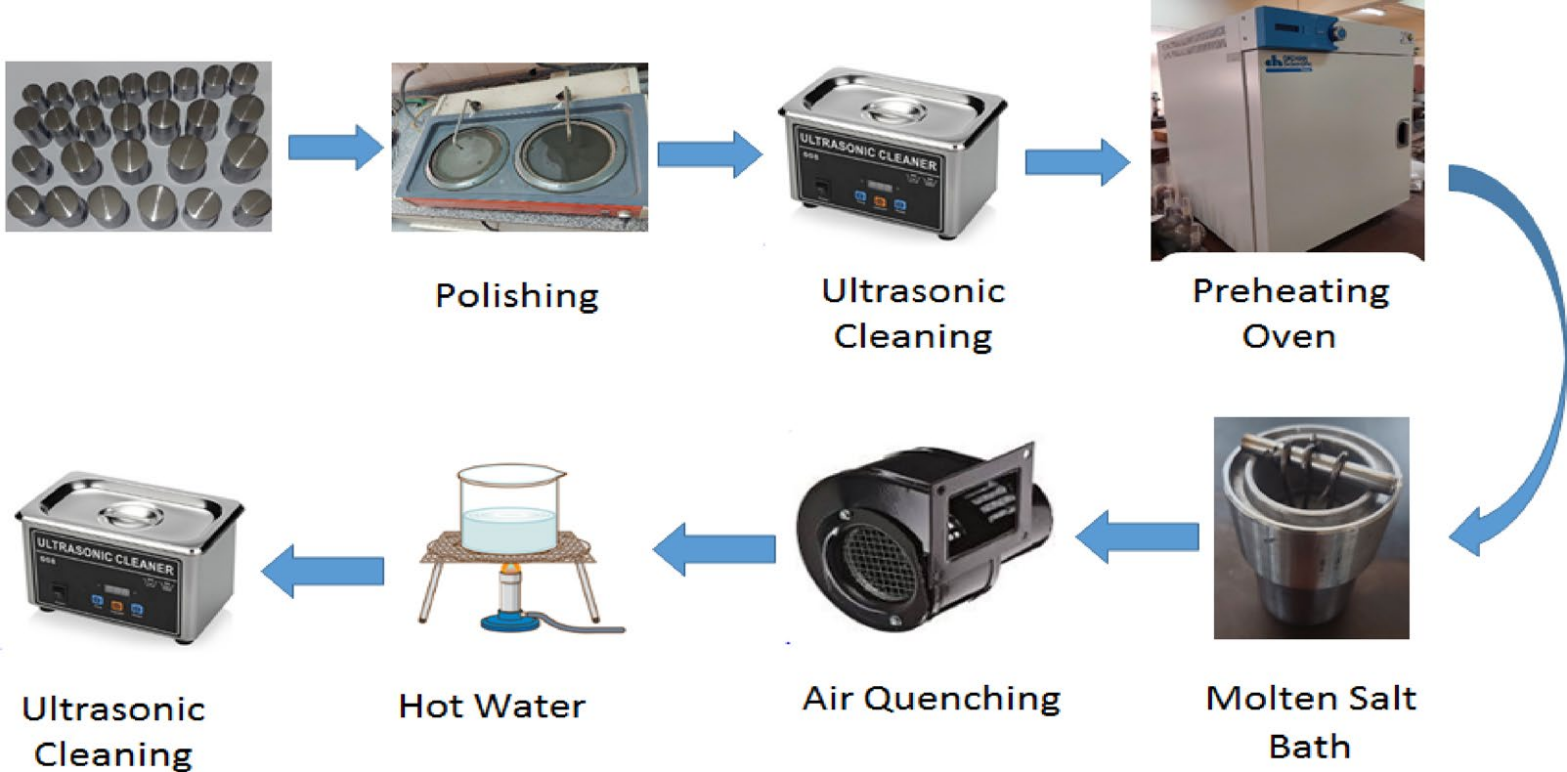
The steps of sample preparation and electrochemical boriding process.

Following the EB treatment, the specimens were taken out of the molten bath and immediately cooled in air. They were then washed in hot water and ultrasonically cleaned for 20 min to remove any adhered materials. A detailed overview of the sample preparation and testing process is provided in Fig. 2.

### Metallographic, wear and hardness examinations

The cross-sections of the specimens were analyzed using a light microscope. Prior to analysis, the specimens were mounted in Bakelite, progressively ground with silicon carbide papers up to 1200 grit, and polished on cloth using 1 µm and 0.3 µm alumina suspensions. Etching was carried out with Nital 2% solution. The specimens were further characterized using scanning electron microscopy (SEM) and energy dispersive X-ray spectroscopy (EDS) to examine the structure, morphology, and composition of the coatings. Additionally, X-ray diffraction (XRD) was performed using a Bruker D6 diffractometer with Cu Kα radiation in the angular range of 10°–100° and a step size of 0.02°. The microhardness profile of the coating and substrate was measured across the coating/substrate according to ASTM E384 standard using a BUEHLER-1600 hardness tester with a maximum load of 50 g and a dwell time of 10 s.

Dry sliding wear tests for the samples were conducted at room temperature using a Ducom TR20LE pin-on-disk wear testing machine. The test involved a sliding velocity of 45 rpm for a total sliding distance of 450 m under a fixed load of 30 N. The pins for the test specimens were prepared and boronized as described earlier, while the disc material consisted of hardened and tempered AISI H13 steel (560 HV_0.1_) coated with a DLC (a-C:H) layer. The pins were cylindrical, with a diameter of 5 mm and a length of 30 mm, and were designed according to ASTM standard G99-05.

## Results and discussion

### Characterization of the coatings

**Fig. 3:**
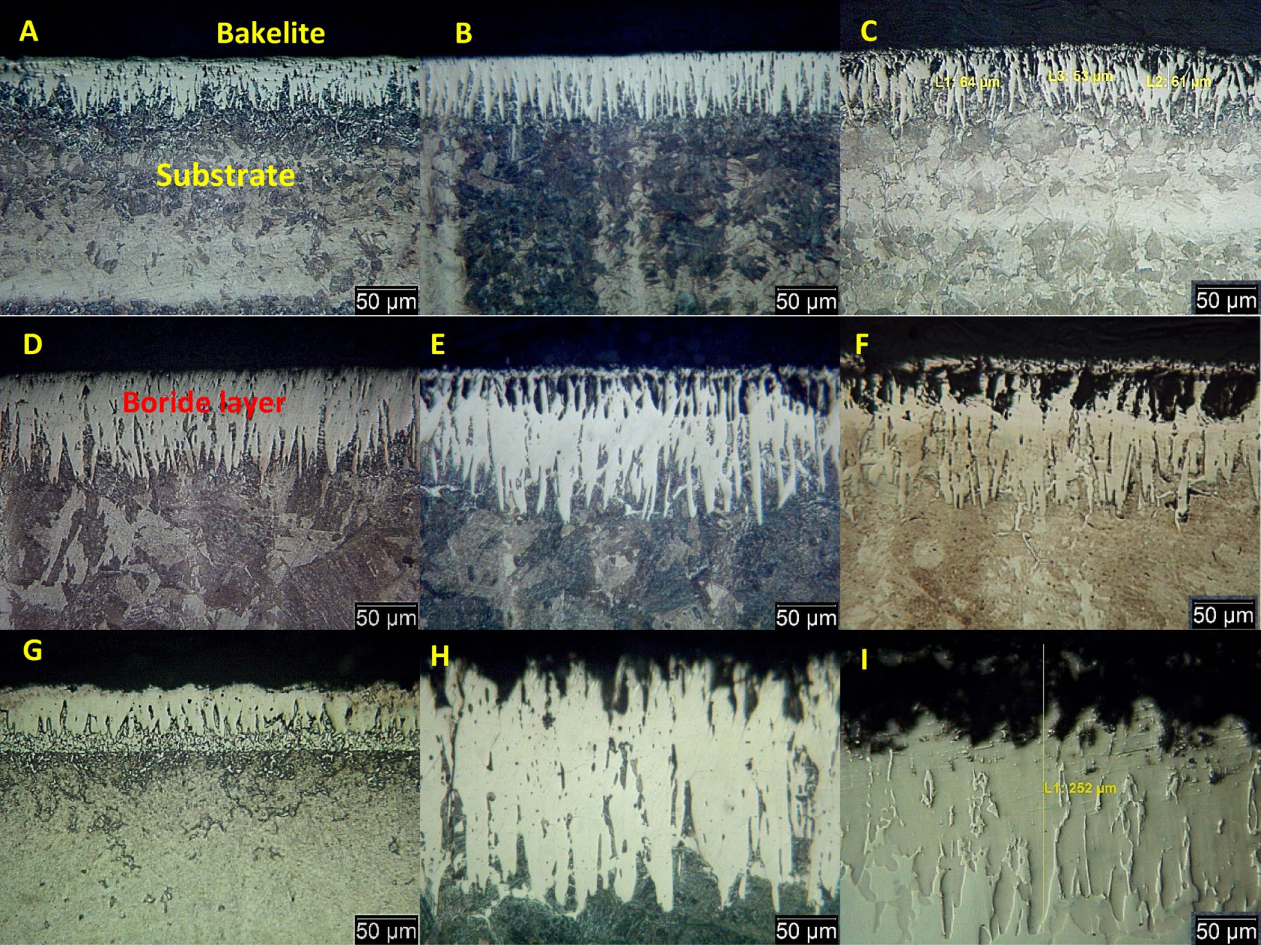
Light microscopy (LM) images of the cross-section of boride AISI H13 tool steel at varying durations (2, 4, and 6 h) and temperatures (850, 950, and 1050 °C), etched with 2% Nital. Specimens (A–I) correspond to those listed in Table 3.

The microstructures of boride samples at three different temperatures 850, 950, and 1050 °C for holding time 2, 4, and 6 h at each temperature degree, are shown in Fig. 3a–i. It is clear that in Fig. 3a–c at 850 °C, there is one layer that increases in thickness with time from 42 to 59 µm. In Fig. 3d and e there is one layer increasing with time from 93 to 110 µm. In Fig. 3f at 950 °C there are two-layers with a thickness of 135 µm. In Fig. 3g–i at 1050 °C there are two-layers increasing with time from 200 to 252 µm.

As shown in Fig. 3, increasing the boronizing temperature from 850 °C to 950 °C results in a more continuous and compact boronizing layer. However, when the temperature further rises from 950 °C to 1050 °C, and with a prolonged holding time of up to 6 h at all temperatures, holes begin to form in the boronizing layer. Notably, when the boronizing temperature exceeds 980 °C, these holes appear in the outermost layer of the boronizing structure. This phenomenon occurs because excessively high temperatures accelerate the diffusion of boron atoms, hindering their ability to form a continuous boronizing layer with surface Fe atoms^30^.

Additionally, the higher temperature causes more gas to escape from the infiltrated layer, leading to the formation of holes and a less dense surface structure. These findings indicate that a boronizing temperature of 950 °C is an optimal choice for the boronizing process.

The boride layers formed on AISI H13 steel under all boriding conditions exhibited a characteristic saw-tooth morphology at the interface with the substrate. This morphology results from the anisotropic diffusion of boron atoms and the crystallographic orientation of the substrate during the formation of Fe_2_B. A distinct transition zone was observed between the compact Fe_2_B layer and the underlying substrate, as confirmed by SEM imaging and EDS line scans. These findings are consistent with previous studies on boride tool steels. Genel et al.^18^, Özbek et al.^15^, and Morón et al.^22^ also reported similar saw-tooth morphologies in boride AISI H13 and other alloy steels, where the interface becomes more pronounced with increasing boriding temperature and time. Additionally, Nair et al.^21^ emphasized the influence of alloying elements such as Cr, Mo, and V on the diffusion profile and layer morphology. The average thickness of the boride layers, summarized in Table 3, shows a direct correlation with increasing temperature and time, consistent with parabolic growth kinetics reported in the literatures^31–33^.

**Table 3:** Experimental values of the entire boride layer thickness.

Specimens codes	Exp. Cond	Layer thickness (µm)
A	850 °C–2 h	42 ± 3.2
B	850 °C–4 h	47 ± 4.5
C	850 °C–6 h	59 ± 5.6
D	950 °C–2 h	93 ± 6.7
E	950 °C–4 h	110 ± 7.6
F	950 °C–6 h	135 ± 8.4
G	1050 °C–2 h	200 ± 11.7
H	1050 °C–4 h	218 ± 13.4
I	1050 °C–6 h	252 ± 15.8

**Fig. 4:**
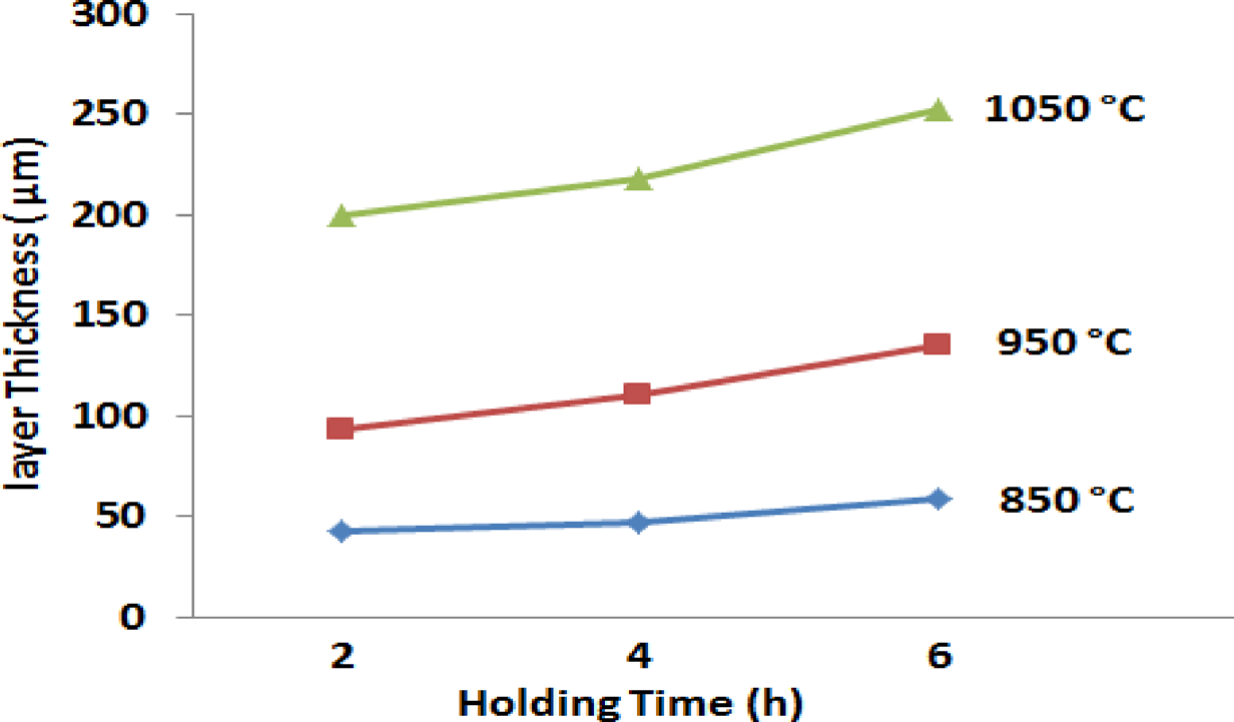
The thickness of iron boride coatings on AISI H13 steel as a function of holding time at various temperatures (850, 950, and 1050 °C).

**Fig. 5:**
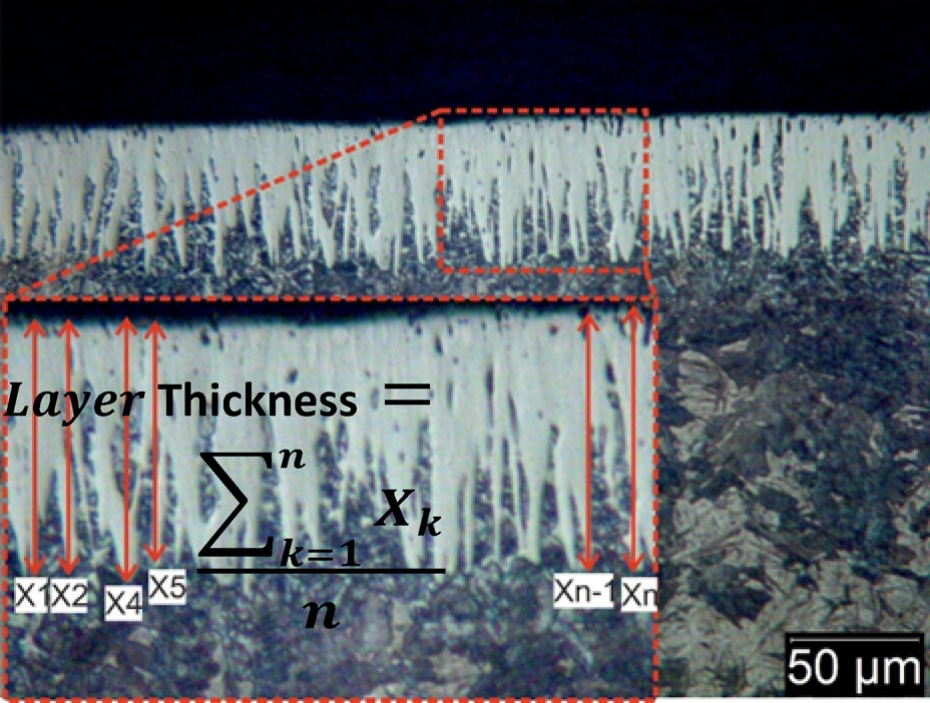
Schematic illustration of boride layer thickness measurement procedure.

Figure 4 illustrates the impact of coating temperature and holding time on the thickness of the boride layer. It was observed that increasing the holding time to 6 h and raising the process temperature to 1050 °C resulted in a thicker boride layer. The relationship between the depth of the boride layer and the diffusion holding time follows an approximately parabolic curve, the thickness of the boronized layer was determined in accordance with the procedure outlined by Türkmen^34^, as shown in Fig. 5.

**Fig. 6:**
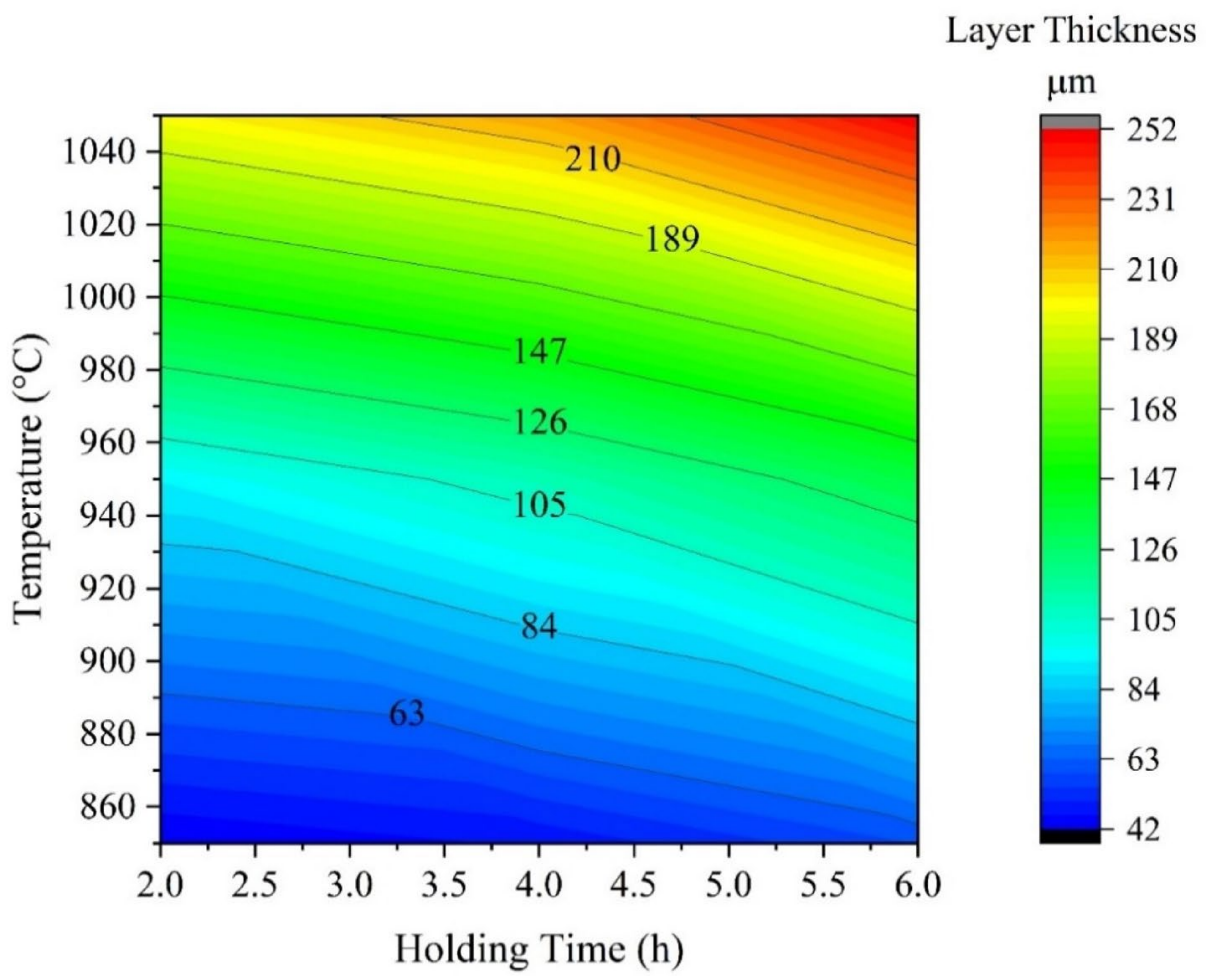
Contour diagram of boride layer thickness of coated AISI H13 depending on process time and temperature.

A contour diagram (Fig. 6) obtained from (Fig. 4) displays the parameters of the process (holding time and temperature) for a predetermined layer thickness for industrial applications of boride AISI H13 steel. In general, thinner layers are suitable for protection against adhesive wear, as encountered in chipless forming operations and metal stamping dies, while thicker layers are preferable for resisting abrasive wear, such as in extrusion tooling for plastics containing abrasive fillers and in pressing tools used in the ceramic industry. We can predict the thickness of the boride layer by using the time and temperature process diagram (Fig. 6). We can use this figure for two goals, firstly, to deduce the value of the time and temperature in obtaining a predetermined coating thickness of layers. The other purpose is to predict the coating layer thickness with respect to time and temperature (process parameters)^5^.

**Fig. 7:**
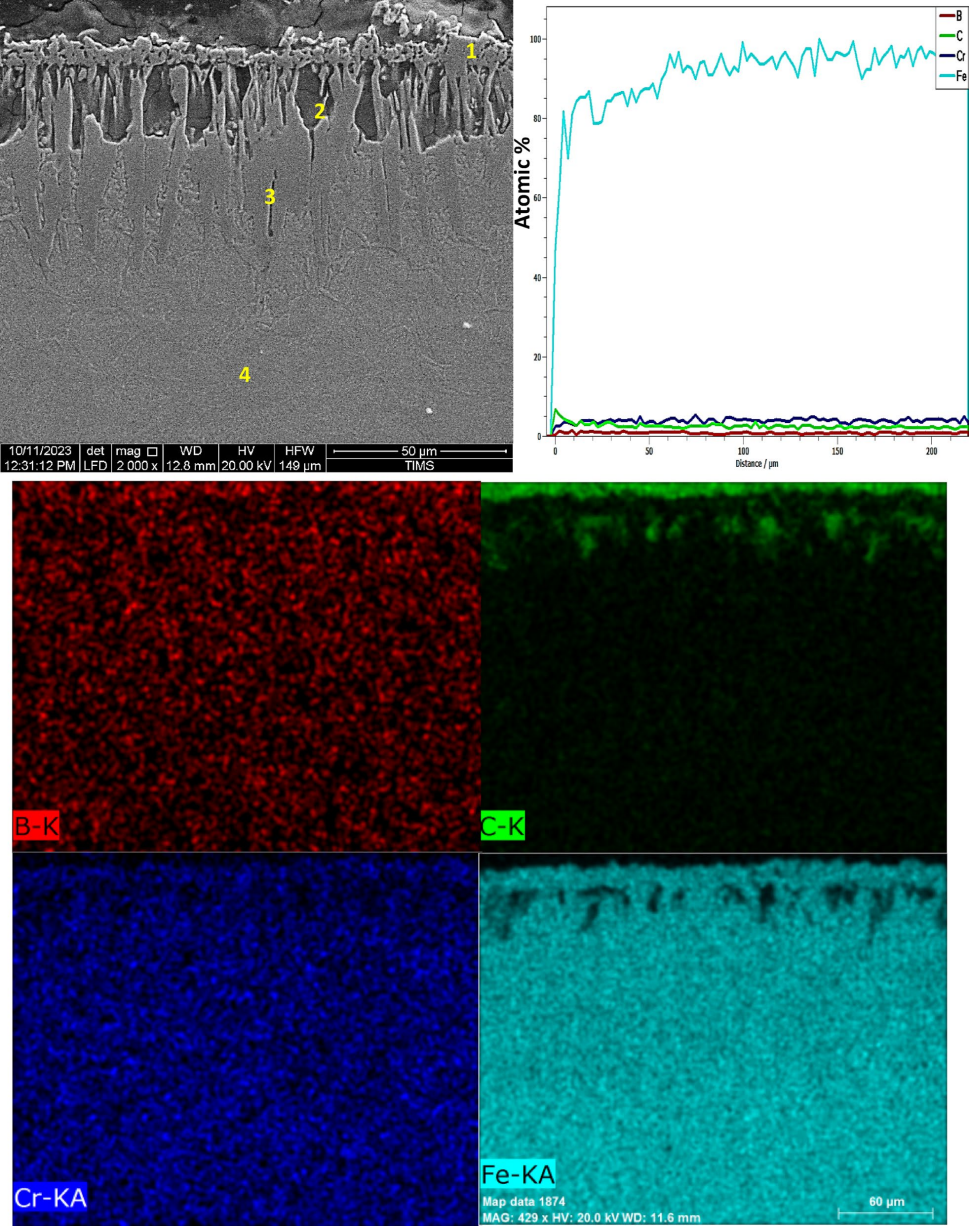
The cross-sectional SEM image, along with line-scan and mapping analysis, of iron boride coatings on AISI H13 tool steel at 850 °C for 6 h.

**Fig. 8:**
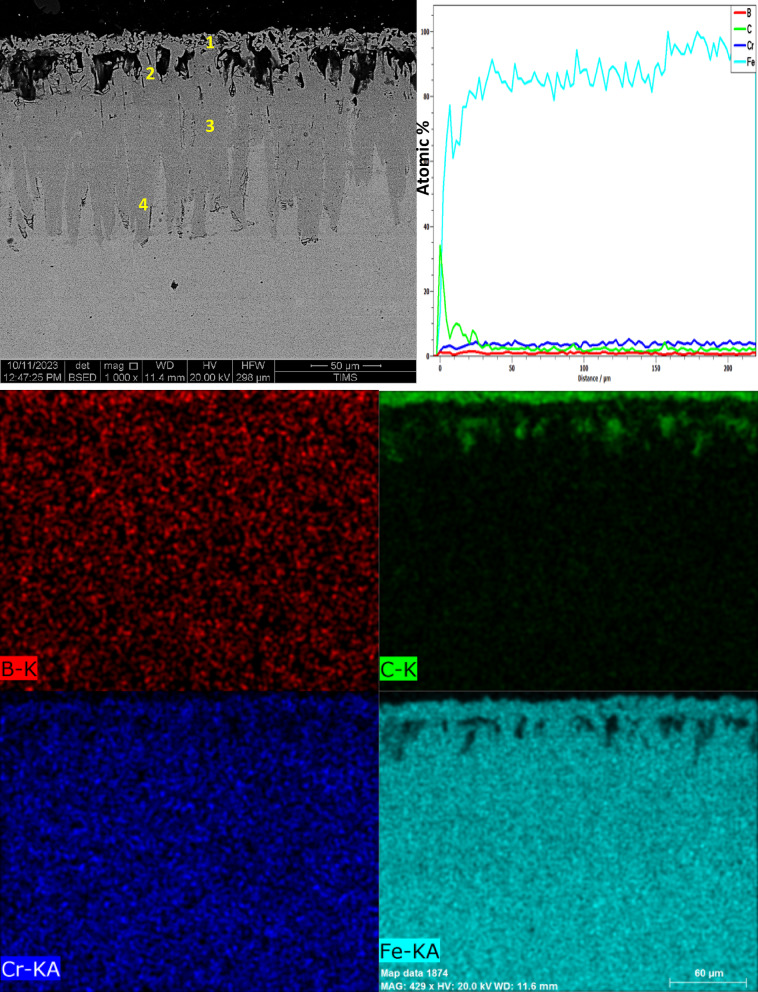
The cross-sectional SEM image, along with line-scan and mapping analysis, of iron boride coatings on AISI H13 tool steel at 950 °C for 6 h.

**Fig. 9:**
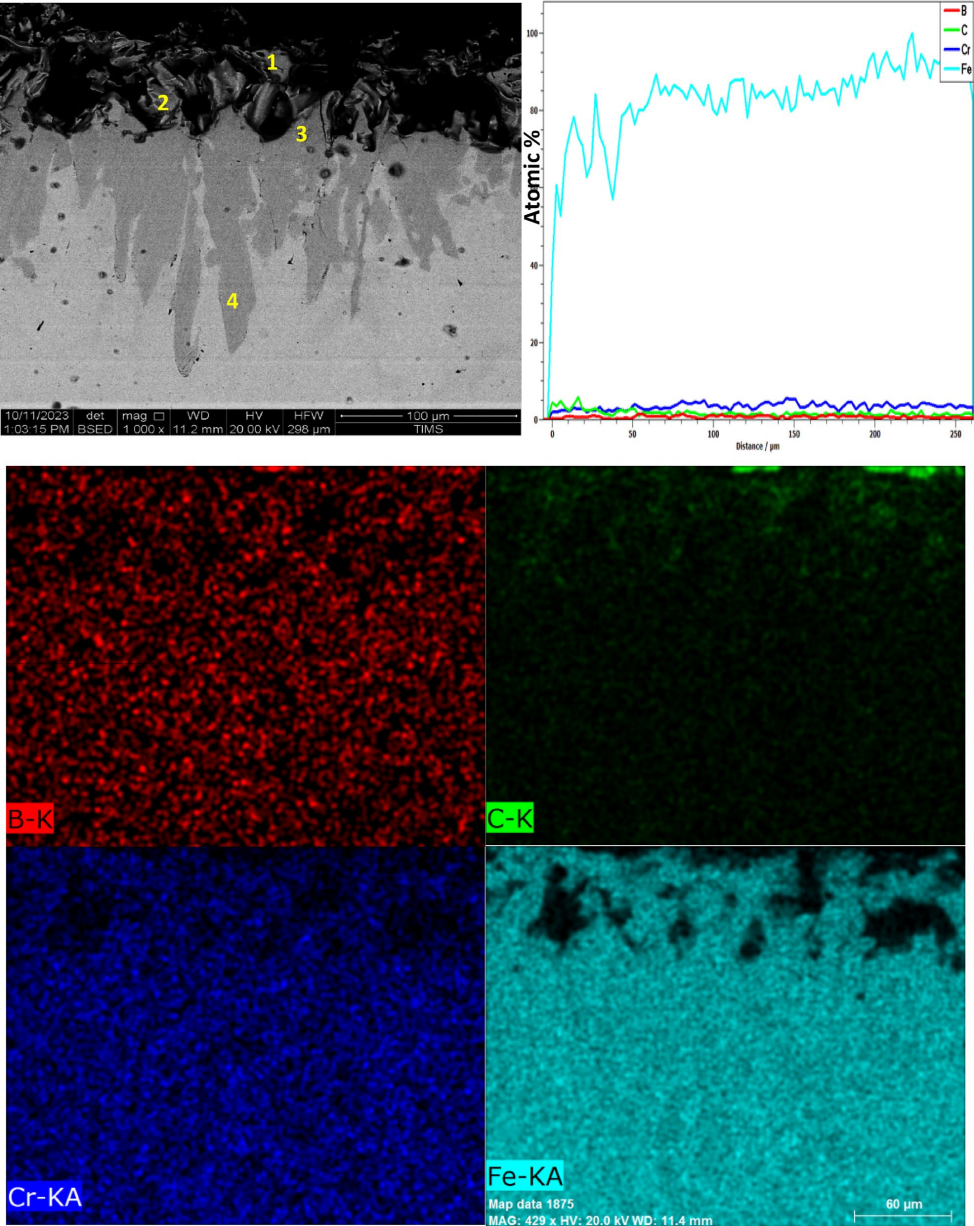
The cross-sectional SEM image, along with line-scan and mapping analysis, of iron boride coatings on AISI H13 tool steel at 1050 °C for 6 h.

SEM images of the cross-sections of three boronized specimens at 850, 950, and 1050 °C for 6 h (Figs. 7, 8, 9) show that the boride layers are dense and homogeneous.

The coatings are continuous and compact under all boriding conditions. A characteristic saw-tooth morphology is observed at the interface between the boride layer and the substrate, which is typical for alloy steels such as AISI H13 due to the diffusion behavior of boron and the influence of alloying elements like Cr, V, and Mo.

**Fig. 10:**
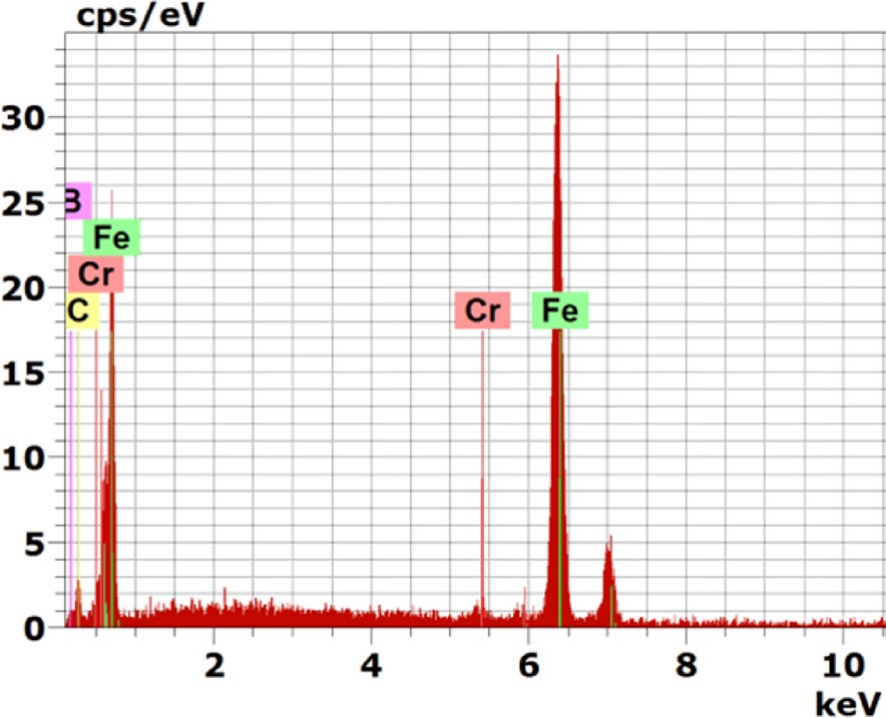
EDS spectrum of the boride sample at point 1 in Fig. 7

**Fig. 11:**
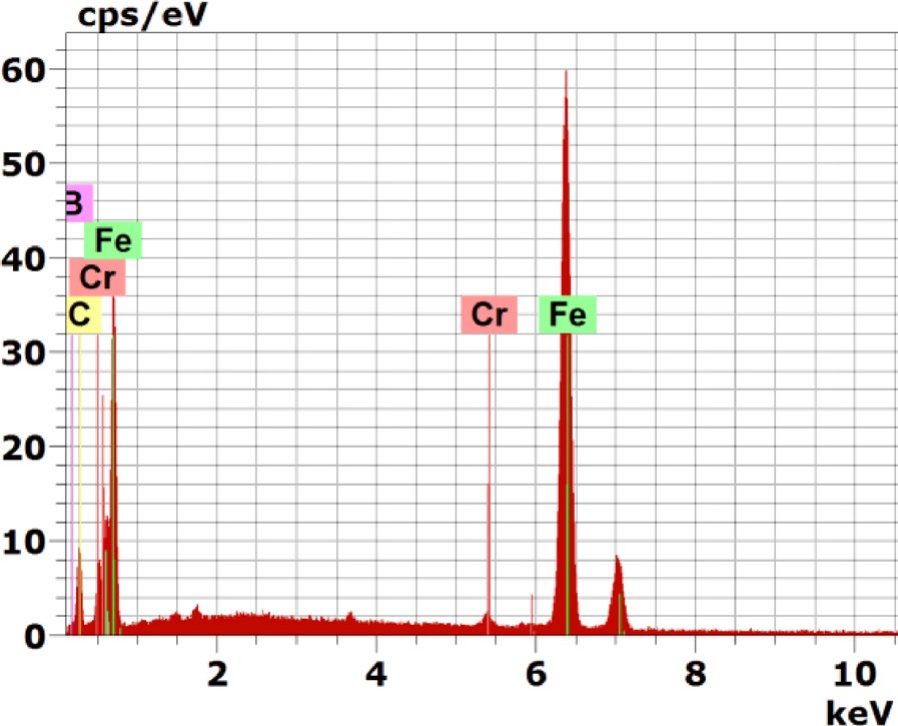
EDS spectrum of the boride sample at point 1 in Fig. 8

**Fig. 12:**
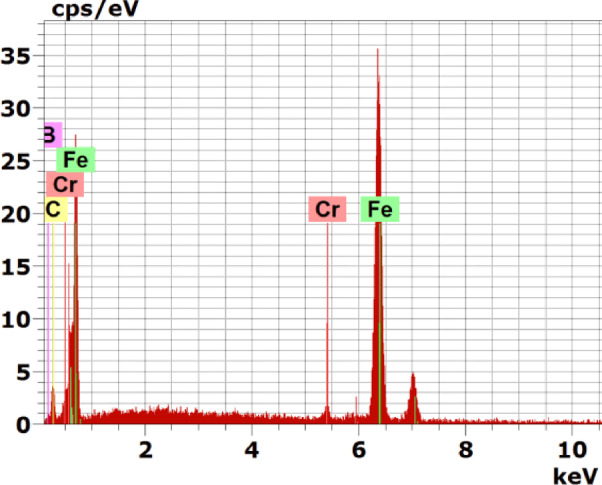
EDS spectrum of the boride sample at point 1 in Fig. 9

It is evident that alloying elements significantly influence the formation of flat interfaces, as observed in certain boride alloyed steels^35–37^. Specifically, a high proportion of alloying elements reduces the active flux of boron atoms and promotes the tendency to flatten the interfaces between the boride layers and the transition zone (Figs. 10, 11, 12).

Figure 7 presents the cross-sectional SEM image, EDS mapping analysis, and line scan of iron boride coatings on AISI H13 tool steel processed at 850 °C for 6 h. The coating consists of two distinct layers: the first (inner) layer, which is free of cracks or porosity, and the second (outer) layer, which exhibits some defects such as cracks. The boron concentration is higher at the top surface of the coating, leading to the formation of the FeB phase. As boron content decreases at the sub-layer, the Fe_2_B phase is formed, followed by a sharp decline in boron concentration at the substrate interface.

**Fig. 13:**
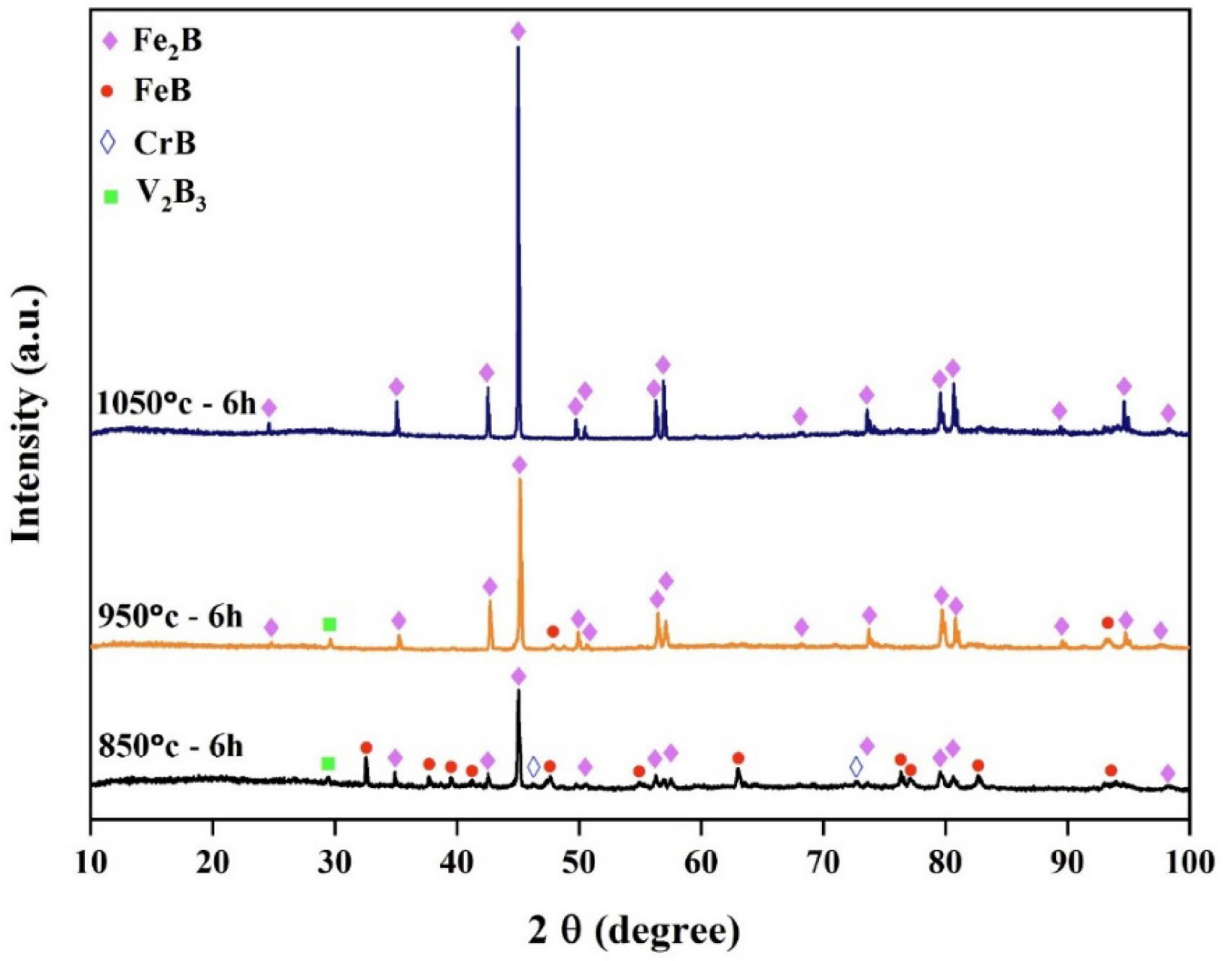
X-ray diffraction patterns from the surface of coated specimens at different temperatures: 850 °C, 950 °C and 1050 °C for 6 h, The COD numbers of the phases, Fe_2_B-1511153, FeB-4003012, CrB-9008948, V_2_B_3_-1510956.

A thin transition zone (TZ) exists between the iron boride layers and the substrate, resulting from the diffusion of elements from the packed powder to the base metal surface and vice versa^38^. This transition zone corresponds to a marked decrease in boron and a sharp increase in iron content, as observed in the EDS line scan. The element composition of the specimen, determined through EDS point analysis, is detailed in Table 4. At point 1, the boron-rich phase corresponds to the formation of the FeB phase. Point 2 represents the Fe_2_B phase, while point 3, near the substrate surface, indicates another region of Fe_2_B phase formation. The EDS analysis aligns with the XRD results (Fig. 13). At point 4, the analysis identifies the composition of the substrate.

**Table 4:** Energy-dispersive X-ray microanalysis EDS (Fig. 7) (wt.%).

Points	B	C	Cr	Fe
1	18.57	14.86	0.85	65.72
2	16.59	17.92	1.52	63.97
3	13.55	8.94	0.92	76.58
4	2.07	13.32	1.01	83.06

Figure 8 presents the cross-sectional SEM image, EDS mapping analysis, and line scan of the iron boride coatings on AISI H13 tool steel treated at 950 °C for 6 h.

The layer coating consists of two distinct layers: the first layer (inner layer) features a uniform thickness and is free from defects such as cracks or porosity, while the second layer (outer layer) contains some defects that appear as surface cracks and porosities. At the top surface of the coating, boron concentration increases, leading to the formation of the FeB phase. As boron concentration decreases, the Fe_2_B phase forms, followed by a sharp drop in boron content at the substrate interface. A thin transition zone (TZ) is visible in the EDS concentration profile between the iron boride layers and the substrate.

The composition of the specimen, as identified through EDS point analysis and presented in Table 5, is as follows: point 1 corresponds to a boron-rich phase indicating the formation of the FeB phase, point 2 indicates the formation of the Fe2B phase, and point 3, near the substrate, also corresponds to the formation of the Fe_2_B phase. The EDS analysis is consistent with the XRD results (Fig. 9). Point 4 reflects the composition of the substrate.

**Table 5:** Energy-dispersive X-ray microanalysis EDS (Fig. 8) (wt.%).

Points	B	C	Cr	Fe
1	14.22	22.18	0.92	62.68
2	15.75	13.95	0.70	69.61
3	16.99	10.66	0.87	71.47
4	15.40	9.03	0.98	74.59

Figure 9 shows the cross-sectional SEM image, mapping analysis, and EDS line scan of the iron boride coatings on AISI H13 tool steel processed at 1050 °C for 6 h. The coating consists of two layers: the first (inner) layer has a uniform thickness and is free of defects such as cracks or porosities, while the second (outer) layer contains some defects, including cracks and porosities.

At the top surface of the coating, the boron concentration increases, leading to the formation of the FeB phase. This is followed by a reduction in boron content, resulting in the formation of the Fe2B phase. Beyond this, a sharp drop in boron content occurs at the substrate. A thin transition zone (TZ) forms between the iron boride layers and the substrate. The elemental composition of the sample, determined through EDS point analysis, is presented in Table 6. Point 1, located at the top surface layer rich in boron, indicates the formation of the Fe_2_B phase. Points 2 to 4 lie within the same outer layer. Figures 10, 11, 12 Energy-dispersive X-ray spectroscopy (EDS) spectra acquired from point 1 in Figs. 7, 8, 9, respectively, for the examined boride samples. The spectra reveal characteristic X-ray emission peaks corresponding to boron (B), carbon (C), chromium (Cr), and iron (Fe), with Fe and Cr detected as the predominant elements.

**Table 6:** Energy-dispersive X-ray microanalysis EDS (Fig. 9) (wt.%).

Points	B	C	Cr	Fe
1	18.08	15.87	1.36	64.69
2	15.61	19.90	0.95	63.54
3	13.05	9.94	1.42	75.59
4	17.37	14.28	0.86	67.49

Figure 13 shows the XRD patterns obtained from the surfaces of electrochemically boronized AISI H13 steel at temperatures of 850, 950 and 1050 °C for 6 h. The XRD analysis indicated the formation of a dual-phase boride layer, which included two types of iron borides (FeB and Fe_2_B), as well as two metallic borides (V_2_B_3_ and CrB), resulting from the chemical affinity of the alloying elements for boron.

The occurrence of other metallic borides on treated AISI H13 steel depends on various boriding conditions, including the nature of the process, the boriding agent, the temperature, and the treatment duration. For example, Kara et al.^25^ identified the CrB phase on the surface of AISI H13 steel boronized at 900 and 950 °C for 4 h using Ekabor 2 powder as the boriding agent. Similarly, Taktak^39^ detected the same CrB phase through XRD analysis when boriding AISI H13 steel via the salt-bath method at 900 °C for 5 h.

The disappearance of the FeB phase in the XRD patterns at elevated boriding temperatures can be attributed to the thermodynamic and kinetic behavior of boride formation on steel substrates. At lower temperatures (e.g., 850 °C), both FeB and Fe_2_B phases are typically observed, with FeB forming as the outermost layer due to the initial high surface boron concentration. However, as the temperature increases (e.g., to 950 °C and 1050 °C), the diffusivity of boron in steel significantly increases, promoting deeper penetration of boron atoms into the substrate and enhancing the formation and growth of the Fe_2_B phase at the expense of the FeB phase.

FeB is thermodynamically less stable than Fe_2_B at higher temperatures and is also more brittle. As boron diffuses inward and reacts with more iron, the FeB layer tends to transform into Fe_2_B according to the reaction: FeB+Fe→Fe_2_B.

This transformation is more favorable at elevated temperatures due to the increased diffusion kinetics and thermodynamic driving force. Consequently, the FeB phase becomes either too thin to be detected by XRD or disappears entirely, leaving Fe_2_B as the dominant boride phase in the coating. This observation is in good agreement with previous studies reporting that higher boriding temperatures and prolonged treatment times favor the exclusive formation of Fe_2_B^40–42^.

### Hardness profile of coatings

**Fig. 14:**
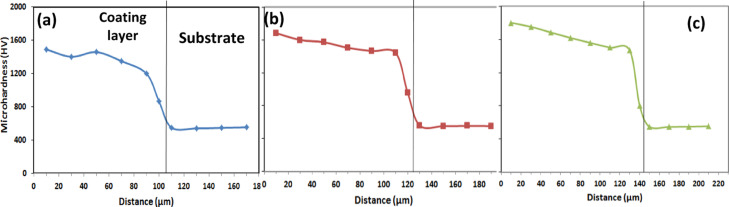
Shows the microhardness profile of the coating/substrate of coated specimens treated at 850 °C with varying holding times: (**a**) 2 h, (**b**) 4 h, and (**c**) 6 h.

**Fig. 15:**
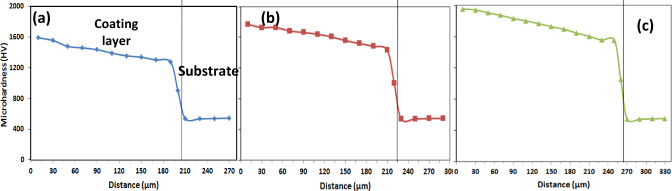
Shows the microhardness profile of the coating/substrate of coated specimens treated at 950 °C with varying holding times: (**a**) 2 h, (**b**) 4 h, and (**c**) 6 h.

**Fig. 16:**
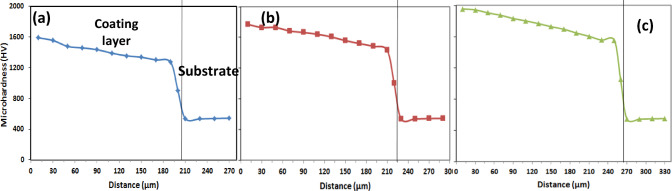
Shows the microhardness profile of the coating/substrate of coated specimens treated at 1050 °C with varying holding times: (**a**) 2 h, (**b**) 4 h, and (**c**) 6 h.

Figures 14, 15, 16 and Table 7 display the microhardness values measured across the cross-sections of the boronized layers, which range from 1300 to 2000 HV_0.05_, depending on the boriding conditions. Specifically, at 1050 °C for 4 and 6 h, the microhardness values were recorded as 1770 and 1956 HV_0.05_, respectively.

**Table 7:** Microhardness results.

Exp. No	Microhardness HV_0.05_
AISI H13 hardened	543 ± 25
A	1343 ± 62
B	1413 ± 70
C	1560 ± 82
D	1490 ± 67
E	1683 ± 78
F	1803 ± 85
G	1590 ± 82
H	1770 ± 75
I	1956 ± 67

A similar trend was observed in the microhardness values obtained at 950 °C for 4 and 6 h. The slight variations in these values can be attributed to the projected contact area at peak load and the extent of the plastic zone beneath the indenter, as reflected in the micro-indentation curves. It was also noted that the microhardness values decreased with increasing diffusion depth, reaching 543 HV_0.05_ in the matrix. These results are consistent with the findings reported by Kara et al.^25^ on boride AISI H13 steels.

The observed differences in surface hardness among the boride samples are primarily attributed to the growth kinetics and phase composition of the formed boride layer. At higher boriding temperatures and longer durations, the diffusion of boron atoms increases, leading to the formation of thicker and more continuous Fe_2_B layers, which are inherently harder than the underlying steel. As a result, samples treated at 1050 °C for 6 h exhibited the highest surface hardness (1956 ± 67 HV_0.05_), while those treated at lower temperatures or shorter durations showed comparatively lower values. Additionally, the microstructural morphology influences hardness; a denser and more uniform saw-tooth Fe_2_B layer enhances resistance to plastic deformation. The presence of alloying elements such as Cr, Mo, and V also plays a role. These elements may form complex borides or affect the boron diffusion rate by altering the thermodynamic and kinetic conditions of the boriding reaction. Cr, in particular, is known to stabilize the Fe_2_B phase and increase the hardness of the layer. Finally, residual stresses induced by the difference in thermal expansion coefficients between the boride layer and the substrate can locally influence microhardness measurements. These combined effects contribute to the variation in hardness values observed among the different boriding conditions.

### Tribological behavior of boronized layer

Before conducting the wear test, both the borided and unborided sample surfaces were prepared to a standard initial surface quality through sanding and polishing. The surface roughness measurements for these samples are presented in Table 8. Analyzing the arithmetic mean of the roughness values (Ra) reveals that the results are relatively similar. The Ra value for the borided samples was measured at the range from 0.72 to 2.27 µm, while the unborided samples had a value of 0.06 µm.

**Table 8:** Surface Roughness of unboride and boride H13.

Specimens codes	Exp. Cond	Roughness (Ra µm)
	AISI H13	0.06
A	850 °C–2 h	2.27
B	850 °C–4 h	2.15
C	850 °C–6 h	2.09
D	950 °C–2 h	1.68
E	950 °C–4 h	1.70
F	950 °C–6 h	0.72
G	1050 °C–2 h	1.71
H	1050 °C–4 h	1.58
I	1050 °C–6 h	1.28

**Fig. 17:**
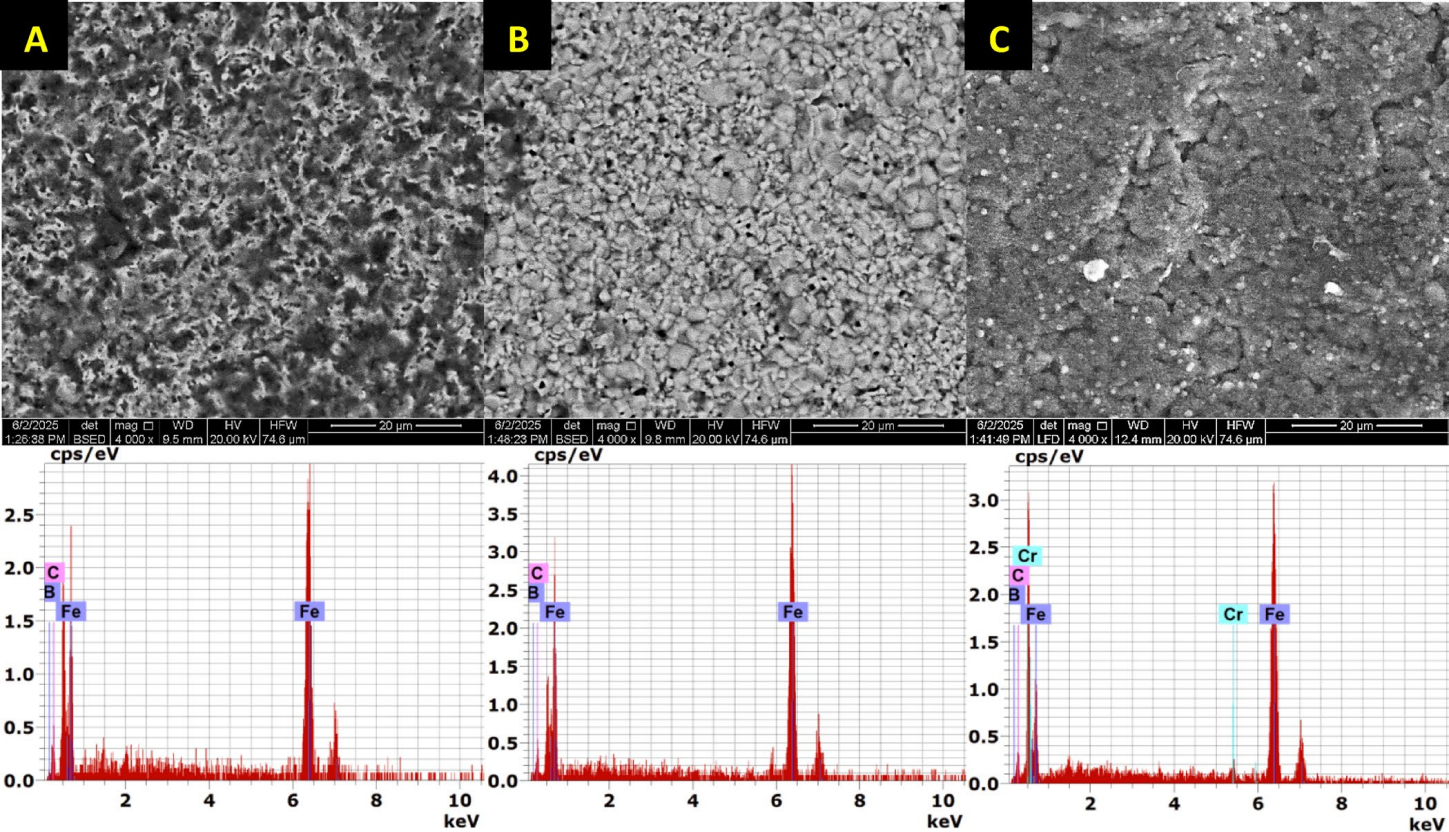
SEM image of surface morphology and EDS analysis of borided samples (**a**) 850 °C–6 h, (**b**) 950 °C–6 h and (**c**) 1050 °C, 6 h.

The surface morphologies of the samples were examined using SEM images taken before the wear test, as shown in Fig. 17. EDS analysis of the borided sample surface revealed the boronizing compounds with some amount of Cr. The borided surfaces displayed porosity and roughness. These morphological features can influence surface roughness and, consequently, wear resistance. Therefore, a correlation is evident between surface morphology and roughness values. Prior to the wear test, EDS analysis was conducted to enable both qualitative and quantitative evaluation of the surfaces (Fig. 17). After the wear test, EDS was again performed on the worn regions. The EDS results before and after the test were compared to identify any new elements or changes in the concentrations of existing elements on the sample surfaces.

Tables 9 and 10 illustrate the mass loss of boronized samples during the friction and wear tests. The test conditions included a force of 30 N, a rotational speed of 200 r/min, and a test duration of 60 min, with sample mass loss data recorded every 10 min. As shown in Table 9, the samples boronized at 850 °C exhibited the highest mass loss, while those boronized at 1050 °C experienced the lowest mass loss.

**Table 9:** Mass loss of boronizing specimens, mg.

Specimens codes	Exp. Cond		Mass Loss (mg)	Average mass loss (mg)		COF	Average COF
	AISI H13	a)	23	22.3 ± 2.08	a)	0.1	0.21 ± 0.115
		b)	24		b)	0.33	
		c)	20		c)	0.2	
A	850 °C–2 h	a)	10	9.2 ± 1.93	a)	0.13	0.16 ± 0.030
		b)	7		b)	0.16	
		c)	10.6		c)	0.19	
B	850 °C–4 h	a)	7	7.1 ± 0.85	a)	0.12	0.15 ± 0.030
		b)	8		b)	0.18	
		c)	6.3		c)	0.15	
C	850 °C–6 h	a)	6	5.7 ± 1.47	a)	0.14	0.13 ± 0.010
		b)	7		b)	0.12	
		c)	4.1		c)	0.13	
D	950 °C–2 h	a)	8	6.9 ± 1.15	a)	0.15	0.14 ± 0.012
		b)	7		b)	0.13	
		c)	5.7		c)	0.15	
E	950 °C–4 h	a)	5	4.8 ± 1.31	a)	0.12	0.11 ± 0.010
		b)	6		b)	0.1	
		c)	3.4		c)	0.11	
F	950 °C–6 h	a)	2	2.9 ± 0.85	a)	0.1	0.09 ± 0.010
		b)	3.7		b)	0.08	
		c)	3		c)	0.09	
G	1050 °C–2 h	a)	5	5.5 ± 0.50	a)	0.12	0.12 ± 0.020
		b)	6		b)	0.1	
		c)	5.5		c)	0.14	
H	1050 °C–4 h	a)	2	2.6 ± 0.53	a)	0.08	0.08 ± 0.010
		b)	3		b)	0.09	
		c)	2.8		c)	0.07	
I	1050 °C–6 h	a)	1.5	1.4 ± 0.10	a)	0.06	0.06 ± 0.010
		b)	1.3		b)	0.07	
		c)	1.4		c)	0.05

**Table 10:** Wear loss of unboride and boride H13 tool steels after the wear testing.

Specimens codes	Exp. Cond	Mass Loss (mg)	Average volumetric wear loss (mm3) × 10^−6^	Specific wear rate (mm^3^/Nm) × 10^−6^
Untreated	AISI H13	22.3 ± 2.08	2,858,970 ± 266,666	211.776 ± 19.7
A	850 °C–2 h	9.2 ± 1.93	1,179,487.18 ± 247,435	87.369 ± 18.3
B	850 °C–4 h	7.1 ± 0.85	910,256.41 ± 108,974	67.427 ± 8
C	850 °C–6 h	5.7 ± 1.47	730,769.23 ± 188,461	54.131 ± 13.9
D	950 °C–2 h	6.9 ± 1.15	884,615.38 ± 147,435	65.527 ± 10.9
E	950 °C–4 h	4.8 ± 1.31	615,384.62 ± 167,948	45.584 ± 12.4
F	950 °C–6 h	2.9 ± 0.85	371,794.87 ± 108,974	27.540 ± 8
G	1050 °C–2 h	5.5 ± 0.50	705,128.21 ± 64,102	52.232 ± 4.7
H	1050 °C–4 h	2.6 ± 0.53	333,333.33 ± 67,948	24.700 ± 5
I	1050◦C–6 h	1.4 ± 0.10	179,487.18 ± 12,820	13.300 ± 0.9

**Fig. 18:**
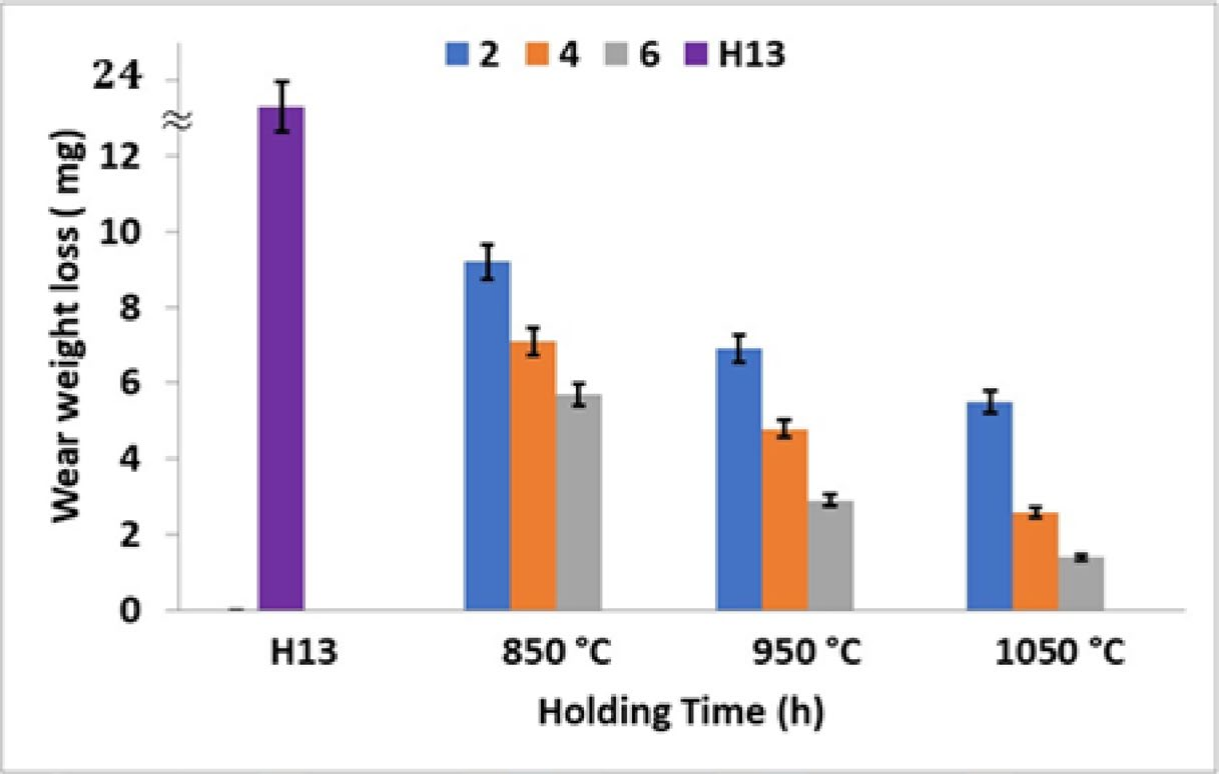
Weight loss of the untreated H13 and boride specimens.

Figure 18 shows the wear loss values for both coated and uncoated samples at various temperatures and immersion times following the wear test. The weight loss of the coatings formed at 850 °C was slightly lower than that of the uncoated sample. However, coated samples at 950 and 1050 °C exhibited significantly improved wear resistance compared to the uncoated sample. Among these, the samples treated at 1050 °C demonstrated the highest wear resistance.

Similarly, samples immersed in the molten salt bath for longer durations showed further improvements in wear resistance. This enhancement is attributed to the increased surface hardness and thickness of the coating layer, which positively influence wear performance. Higher hardness reduces the penetration of the abrasive pin and wear debris into the substrate, as noted by Günen et al.^28^.

After 60 min of wear testing, the mass loss of hardened AISI H13 steel (untreated) was 22.3 mg, compared to 1.3 mg for samples boronized at 1050 °C for 6 h and 5.7 mg for samples boronized at 850 °C for 6 h. Specifically, the mass loss of uncoated AISI H13 was 3.7 times greater than that of samples boronized at 1050 °C for 6 h and 2.4 times greater than boronized samples at 850 °C for 6 h.

This improvement in wear resistance can be attributed to the increased temperature, which enhances the activity of boron atoms. The higher temperature facilitates the diffusion of active boron atoms into the material, thereby increasing the efficiency of the boronizing process. As a result, the quality of the boronized layer improves, leading to superior wear resistance.

#### Wear and coefficient of friction

**Fig. 19:**
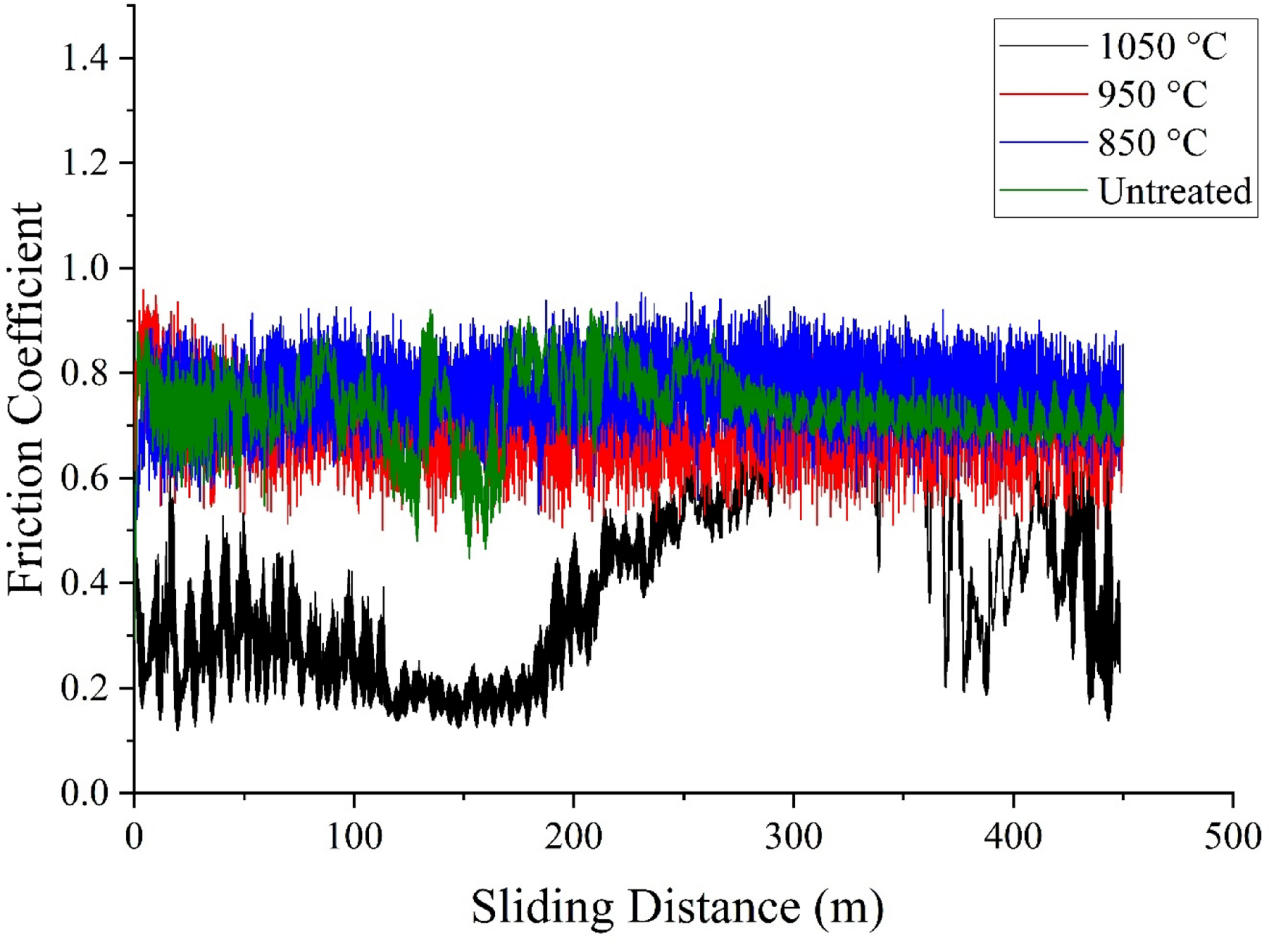
COF versus load for untreated and boride AISI H13 tool steel at untreated, 850 °C, 950 °C and 1050 °C for 6 h.

Figure 19 displays graphs of the coefficient of friction (COF) as a function of sliding distance from wear tests performed on both untreated AISI H13 substrate and coated samples. The untreated AISI H13 shows a rapid increase in COF during the first 130 m of sliding, followed by a stabilized trend. This behavior suggests that the applied load may have induced deformation hardening in the material. This is supported by the observed increase in surface hardness, which measured 360 HV before the wear test and 485 HV afterward. Another contributing factor could be the rise in local temperature during sliding, promoting the formation of low-friction adhesive oxide films due to the tests being conducted in air^28^.

The COF curves for samples coated at 850 °C are shown in Fig. 19. For a 2 h immersion, the COF values were similar to those of untreated AISI H13, likely because only a small number of iron boride nuclei formed at this stage. Increasing the immersion time to 4 and 6 h resulted in a greater formation of iron boride nuclei, thereby reducing COF values. However, significant fluctuations in COF were observed throughout the test period due to the non-uniform and porous nature of the coatings formed at 850 °C.

For the sample coated at 850 °C for 6 h, the wear curve showed an initial increase in COF during the first 80 m, followed by a slight rise up to 133 m, and then a sudden decrease. This increase in COF is likely attributed to the destruction of iron boride nuclei and the iron boride solid solution.

Figures 19 also display the COF graphs for samples coated at 950 and 1050 °C, respectively. Across all cases, the coated samples exhibit lower COF values compared to uncoated AISI H13, consistent with findings reported by other researchers^43^. The reduced friction coefficients of iron boride coatings, compared to the substrate, can be attributed to the high hardness of the iron boride phases and the higher carbon content within the coatings^28^. Furthermore, the coatings formed at 950 °C and 1050 °C showed less COF fluctuation, likely due to their uniform and non-porous structure.

It was also observed that as the thermo-reactive deposition TRD temperature and immersion time increased, the average COF values of the coated samples decreased. This is because thicker and harder boride coatings enhance friction performance. As a result, coatings formed at 1050 °C demonstrated superior friction behavior compared to those formed at 850 °C and 950 °C.

During wear testing, the applied load can cause the coatings to break into hard particles, which can act as abrasives between the pin and the surface, leading to an increase in COF throughout the wear test period^44^.

#### Worn surface analysis

**Fig. 20:**
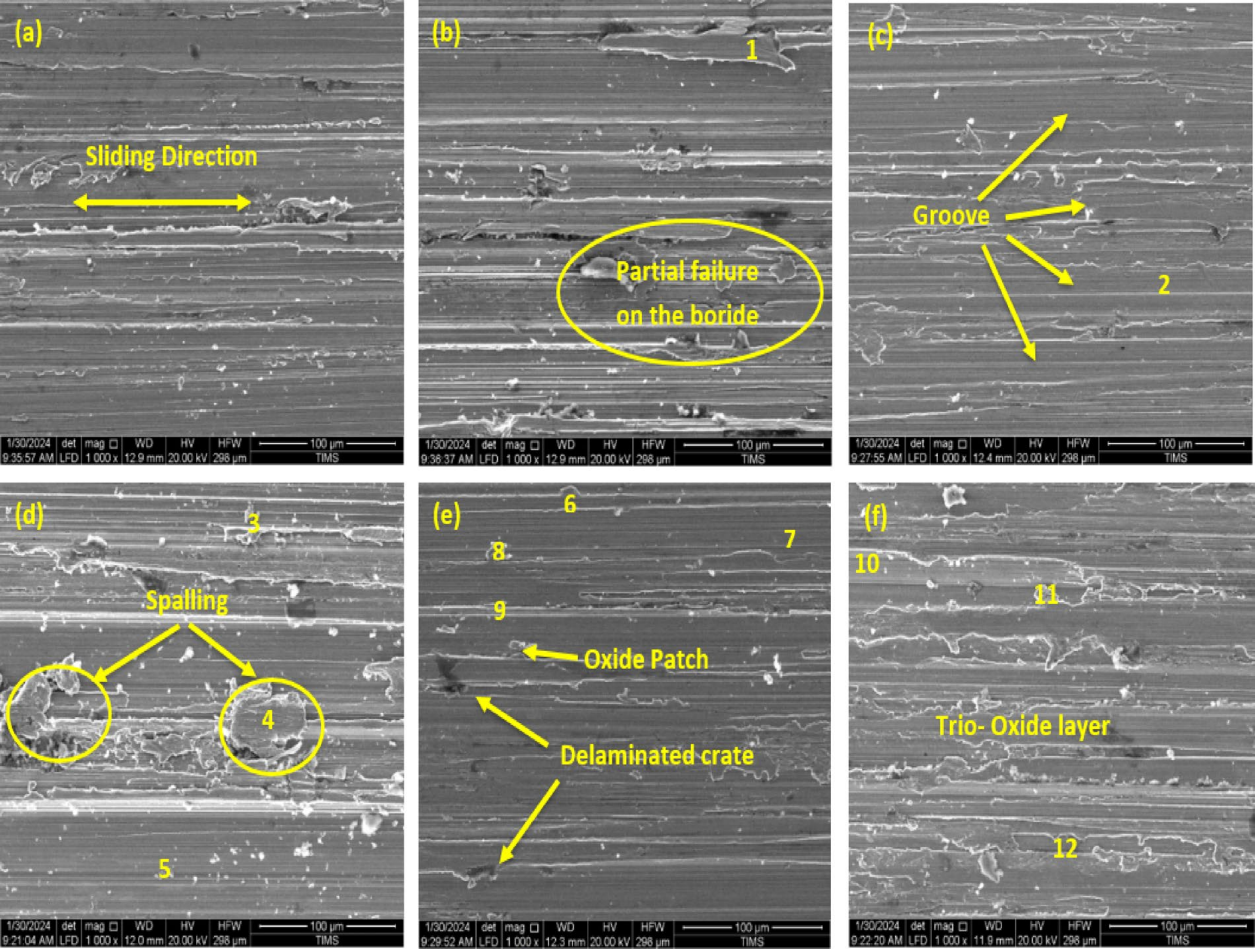
SEM images of the worn surface of boride pins.

Figure 20 displays the SEM micrograph and EDS analysis of the worn surface of the untreated AISI H13 substrate and the boronized samples. The SEM micrograph shows dark and grey regions representing the transfer layer. EDS analysis of the very dark grey region confirms the presence of a significant amount of boron (B). The grey region contains carbon (C) and chromium (Cr), while the brown region indicates iron (Fe). The whitish region predominantly shows iron (Fe) with a low carbon (C) content. In the boronized specimens, EDS analysis reveals boron’s presence, demonstrating a modification of the transfer layer, which consists of a mix of the original carbon film and elements from the counter face. This finding highlights the triboreactivity of the DLC coating with the pin surface. The transfer layer also contains elements such as C, Fe, Cr, and V, which have been reported in the literature. According to Bhushan^45^, the reduction in wear rate is associated with the modification of the transfer film composition under high loads and high speeds. Table 11 provides the elemental composition of the specimen as determined by EDS point analysis. The EDS analysis results presented in Table 11 confirm the presence of oxygen in several analyzed regions, particularly at points corresponding to oxide patches (Fig. 20e and f). The detection of oxygen indicates the formation of surface oxides during the sliding process, which is consistent with the tribological observations. The occurrence of these oxide patches can be attributed to localized frictional heating and oxidation of the boride surface during wear testing. The formation of a thin tribo-oxide layer is known to act as a solid lubricant, reducing direct metallic contact and contributing to the stabilization of the friction coefficient. This behavior aligns with previous reports on boride tool steels, where oxide formation under sliding conditions enhanced wear resistance and reduced adhesive wear.

**Table 11:** Energy-dispersive spectroscopy EDS microanalysis (Fig. 20) (wt.%).

Points	El [wt.%]							
	B	C	Si	V	Cr	Fe	O	Mo
1	3.31	8.26	0.09	0.05	1.08	77.18	9.74	0.29
2	13.09	7.12	0.75	64.44	0.89	9.58	3.49	0.64
3	9.21	0.47	0.03	1.13	80.91	0.27	0.19	7.79
4	16.58	8.24	0.04	0.38	0.82	68.48	4.85	0.61
5	12.33	17.92	0.36	0.53	56.89	0.31	0.99	10.67
6	0.00	10.25	0.88	0.68	4.55	78.68	3.96	1.00
7	11.50	8.79	1.04	0.92	4.03	70.62	1.98	1.12
8	4.27	8.11	0.95	0.55	4.90	76.60	4.36	0.26
9	9.30	9.94	1.01	0.91	3.65	61.32	13.36	0.51
10	1.29	8.63	1.24	2.05	4.78	69.78	10.21	2.19
11	14.23	4.65	1.15	1.05	3.52	67.23	8.17	0.00
12	0.00	11.45	0.82	1.37	10.74	62.39	12.09	1.14

### Boronization kinetics study

The growth kinetics of the boride layer on AISI H13 steel were analyzed by plotting its thickness against the square root of the treatment time at varying temperatures, following the Arrhenius.

equation. The relationship between penetration time, the thickness of the penetration layer, and the growth coefficient can be expressed using Eq. (1)^46^.

1 $${\text{X2}} = {\text{Gt}}\quad {\text{X}} = \surd {\text{G}}*{\text{T}}$$

**Fig. 21:**
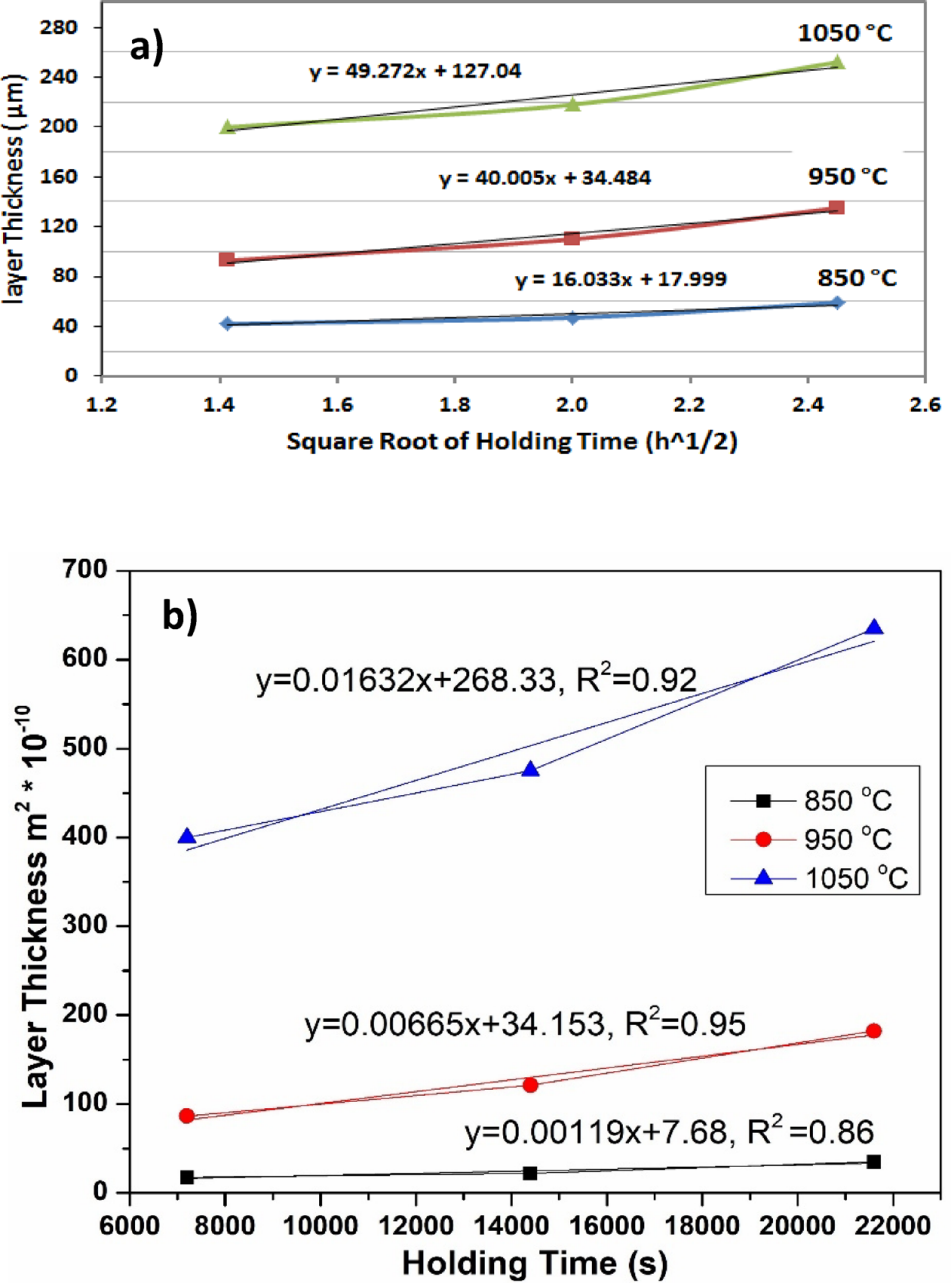
(**a**) Boride layer thickness on AISI H13 versus square root of boriding holding time, (**b**) square of the boride layer thickness versus boriding holding time for various process temperatures 850, 950 and1050 °C.

In this context, X represents the total thickness of the boride layer (µm), G is the diffusion coefficient of boron (m2⋅s − 1), and t is the treatment time (s). The kinetics of boronizing are controlled by the diffusion of boron atoms into the substrate, resulting in the formation of a hard boride layer on the material’s surface. Table 3 lists the experimentally measured layer thicknesses based on the boriding parameters (temperature and treatment time). By plotting the total boride layer thickness as a function of the square root of time (Fig. 21), the growth rate constants (K) at 850, 950, and 1050 °C were determined and are presented in Table 12.

**Table 12:** Experimental parabolic growth rate constants K in the temperature range 850, 950 and 1050 °C.

Temperature °C	Growth rate constant k (m^2^/s)
850	1.19*10^–13^
950	6.65*10^–13^
1050	16.3*10^–13^

The boronizing temperature influences the growth constant (K) of the coatings. It can be calculated using the Arrhenius equation^1,47^.

2 $${\text{K}} = {\text{K}}_{0} *{\text{e}}^{{ - ({\text{Q}}/{\text{RT}})}}$$

In the Arrhenius equation, k_0_ represents the pre-exponential constant (m^2^/s), Q is the activation energy of the process (J/mol), R is the gas constant (J/mol K), and T is the absolute temperature (K). By taking the logarithm of the Arrhenius equation, the following expression is derived:

3 $${\text{lnK}} = {\text{lnK}}_{0} {-}\left( {{\text{Q}}/{\text{R}}*{1}/{\text{T}}} \right)$$

**Fig. 22:**
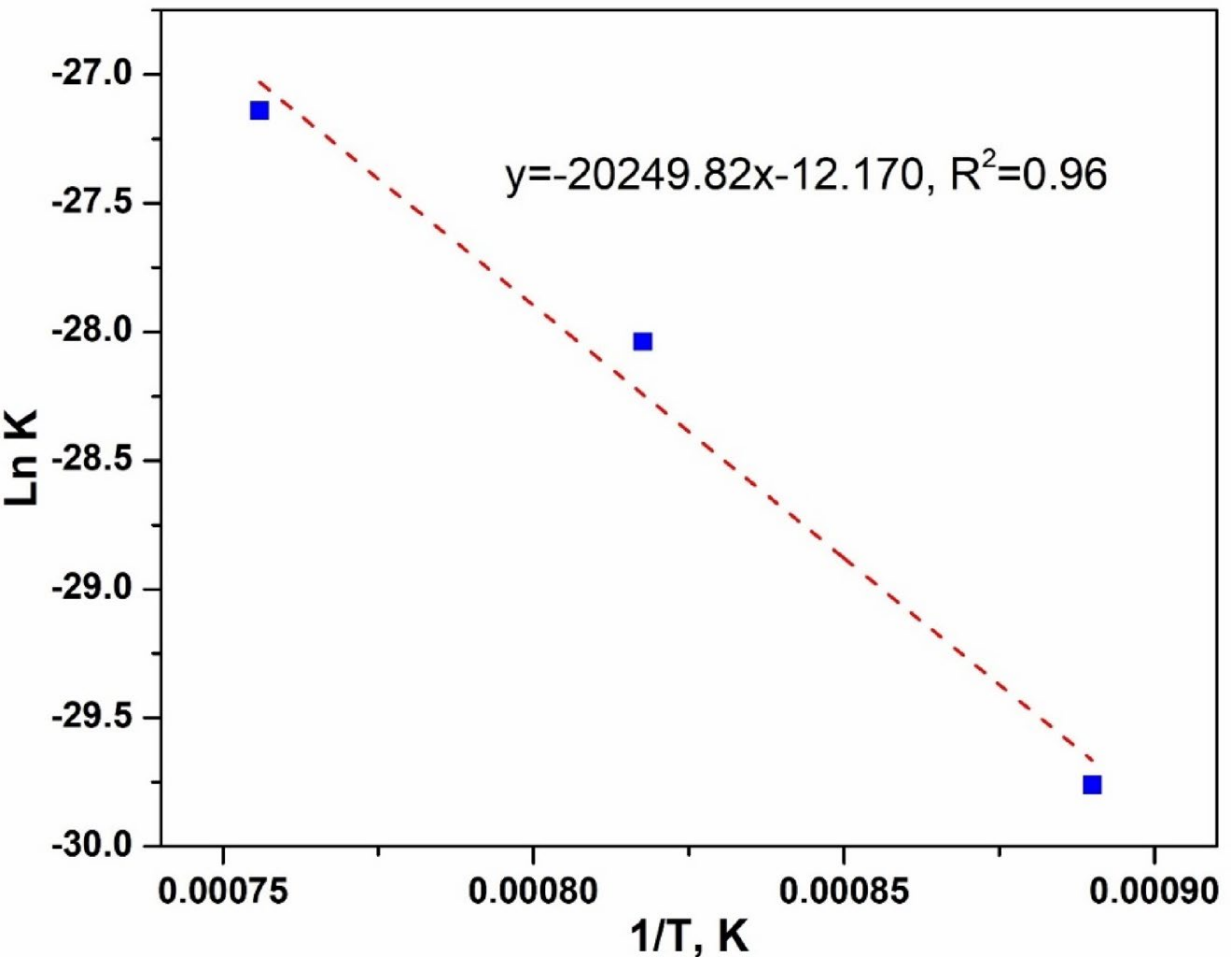
Arrhenius relationship relating the boron diffusion coefficient in the dual phase boride layer to the boriding temperature.

To calculate the values of Q and k_0_, Fig. 22 shows a plot of logarithmic K versus 1/T for iron boride coated AISI H13 tool steel. Based on the data in Fig. 22, the calculated activation energy (Q) is 168.4 kJ/mol for (Fe_2_B+FeB) layers, which is lower than the values measured by Keddam, 232.62 and 240.37 kJ/mol for Fe_2_B and FeB layers formed on AISI H13 during pack boronizing at temperatures between 850 and 1000 °C for durations of 2 to 8 h^24^. Mei et al.^30^ conducted pack boronizing on AISI H13 steel using a powder mixture containing rare earth oxides (CeO_2_) at concentrations ranging from 2 to 6 wt.% within the same temperature range. The activation energy for the FeB+Fe_2_B layers was between 143.16 and 160.70 kJ/mol, which is slightly lower than the value found in this study. Table 13 gives a comparison between the value of activation energy estimated in this work and the values taken from the literature^18,23,32,48,49^ for boronized AISI H13 steels.

**Table 13:** Comparison of the calculated activation energy with the values reported in the literature for boride AISI H13 steels.

Boriding method	Temperature range (°C)	Activation energy (kJ mol^−1^)	References
Powder	800–1000	227.5 (FeB+Fe_2_B) with Nanoboron (NB)284.2 (FeB+Fe_2_B) with Ekabor II	^23^
Paste	900–1000	219.2 (FeB), 189.6 (Fe_2_B)	^48^
Powder	850–950	185.7 (FeB+Fe_2_B)	^49^
Powder	800–1000	186.2 (FeB+Fe_2_B)	^18^
Powder	850–1000	233 (Fe_2_B)	^32^
Electrochemical	850–1050	168.4 (FeB+Fe_2_B)	Present work

The calculated pre-exponential constant K_0_ is 8 *10^–6^ m^2^/s. The following is the derived formula between the parabolic growth rate constant of iron borides coating and temperature:

4 $${\text{K}} = 8 \times 10^{ - 6} {\text{e}}^{{ - 20250/{\text{T}}}}$$

As a result, combining Eq. 1 and 3. The relationship between iron boride layer thickness d and treatment time t can be expressed as follows:

5 $${\text{d}} = \surd {8} \times {1}0^{{ - {6}}} {\text{te}}^{{ - {2}0{25}0/{\text{T}}}}$$

Table 14 presents the correlation between the boron diffusion coefficient and the boriding temperature using the Arrhenius relationship. The table compares the experimental and estimated boride layer thicknesses at different temperatures and holding times. A good agreement is observed between the experimental data and the values estimated using Eq. 5, validating the diffusion-based kinetic model applied for the dual-phase boride layer growth.

**Table 14:** Comparison between experimental and estimated boride layer thicknesses at various boriding conditions based on the Arrhenius diffusion model.

Specimens codes	Exp. Cond	Experimental layer thickness (µm)	Boride layer thickness estimated by Eq. 5 (µm)
A	850 °C–2 h	42 ± 3	41
B	850 °C–4 h	47 ± 4	58
C	850 °C–6 h	59 ± 6	71
D	950 °C–2 h	93 ± 7	86
E	950 °C–4 h	110 ± 8	122
F	950 °C–6 h	135 ± 8	149
G	1050 °C–2 h	200 ± 12	161
H	1050 °C–4 h	218 ± 13	227
I	1050 °C–6 h	252 ± 16	278

## Conclusions

In this study, AISI H13 tool steel was successfully boronized using the electrochemical boriding (EB) technique in a molten borax bath containing ferroboron powder at 850, 950, and 1050 °C for holding times of 2, 4, and 6 h, enabling a detailed assessment of temperature and time effects. The resulting boride layers exhibited either a single-layer Fe_2_B structure or a dual-layer FeB/Fe_2_B structure, with total thicknesses increasing significantly with both temperature and duration, reaching up to 252 µm. XRD analysis confirmed the presence of FeB, Fe_2_B, and transition-metal borides (CrB and V_2_B_3_). The coating–substrate interfaces were smooth and well-bonded, indicating good adhesion.

The EB-treated surfaces demonstrated marked improvements in hardness, with microhardness values between 1343 and 1956 HV_0.05_ compared to 543 HV for the uncoated substrate, and tribological tests showed significantly superior wear resistance, validating the effectiveness of EB for enhancing surface performance under severe operating conditions. Boride layer growth followed a parabolic law, and the activation energy for boron diffusion was calculated as 168.4 kJ/mol, consistent with the influence of alloying elements on boron mobility and the Arrhenius diffusion model. Overall, the substantial hardness increase achieved through EB directly translated into enhanced wear resistance, underscoring the process’s potential to extend the service life of AISI H13 steel components in demanding industrial applications.

## Data Availability

The datasets used and/or analysed during the current study available from the corresponding author on reasonable request.
